# Let-7a induces metabolic reprogramming in breast cancer cells via targeting mitochondrial encoded ND4

**DOI:** 10.1186/s12935-021-02339-3

**Published:** 2021-11-27

**Authors:** Praveen Sharma, Vibhuti Sharma, Tarunveer Singh Ahluwalia, Nilambra Dogra, Santosh Kumar, Sandeep Singh

**Affiliations:** 1 Molecular Medicine Laboratory, Department of Human Genetics and Molecular Medicine, Central University of Punjab, Bathinda, India; 2 Centre for Systems Biology and Bioinformatics, Panjab University, Chandigarh, India; 3 Steno Diabetes Center, Copenhagen, Denmark; 4 Department of Biochemistry, AIIMS, Patna, India

**Keywords:** Mitochondria, Metabolic reprogramming, Mito-miRs, Glycolysis, Cancer

## Abstract

**Background and objectives:**

MicroRNA (miRNA) that translocate from the nucleus to mitochondria are referred to as mitochondrial microRNA (mitomiR). Albeit mitomiRs have been shown to modulate gene expression, their functional impact within mitochondria is unknown. The main objective of this study is to investigate whether the mitochondrial genome is regulated by miR present inside the mitochondria.

**Methods and results:**

Here, we report mitomiR let-7a regulates mitochondrial transcription in breast cancer cells and reprogram the metabolism accordingly. These effects were mediated through the interaction of let-7a with mtDNA, as studied by RNA pull-down assays, altering the activity of Complex I in a cell line-specific manner. Our study, for the first time, identifies the role of mitomiR (let-7a) in regulating the mitochondrial genome by transcriptional repression and its contribution to regulating mitochondrial metabolism of breast cancer cells.

**Conclusion:**

These findings uncover a novel mechanism by which mitomiR regulates mitochondrial transcription.

**Supplementary Information:**

The online version contains supplementary material available at 10.1186/s12935-021-02339-3.

## Introduction

Cancer cells not only use aerobic glycolysis, but instead, they can also utilize intermediates generated during glycolysis and can generate lactic acid and biocarbonic acid [1]. Metabolic reprogramming is the hallmark of cancer as cancer cells alter their metabolism to meet sufficient energy supply to sustain tumor growth and proliferation [2]. Metabolic reprogramming also promotes invasion, metastasis, increased resistance to various therapies, highlighting the role of metabolic rewiring in cellular transformation [3]. Warburg also characterized the metabolic phenotype of cancer cells stating that cancer cells do not entirely depend on glycolysis and, therefore, may shift from oxidative phosphorylation to glycolysis to fulfil anabolic growth [4]. Cellular and metabolic complexity forces cancer cells to reprogram the mitochondrial-mediated metabolic pathways to promote cancer growth, and these modulations, in turn, result in the activation of nuclear signals [5].

Mitochondria are essential organelle involved in regulating various fundamental cellular functions, including maintaining calcium homeostasis, apoptosis, oxidative phosphorylation (OXPHOS) [6]. The Human mtDNA encodes for 13 polypeptides responsible for oxidative phosphorylation, 2rRNAs, and 22tRNAs for mitochondrial protein synthesis [7, 8]. Due to the association of proto-mitochondrial genes with the nucleus throughout the evolution, several mitochondrial proteins are synthesized in the nucleus and get translocated into the mitochondria, but mitochondrial replication is not synchronized with the nuclear replication [9, 10]). Since mitochondria play a major role in cellular respiration and cell death, they have been widely reported to be involved in metabolic alterations in cancer cells [11]. Some reports also indicate that the quality of mitochondria governs the efficacy of anticancer drugs [12]. One such drug, doxorubicin (DOX), is known to induce mitochondrial stress by altering cell signaling, and mitochondrial specific reactive oxygen species (ROS) generation, and membrane potential alteration [13]. There is evidence suggesting that DOX causes mitochondrial dysfunction by inducing mitochondrial localization of critical proteins like p53 [14].

Recent studies indicate a dynamic relationship between mitochondrial function and microRNA (miRNA) activity [9, 15]. miRNAs detected inside the mitochondria (named as mito-miRs) are transcribed in the nucleus and were translocated into the mitochondria [16]. The association of miRNAs with mitochondria raises the possibility that mito-miRs may regulate mitochondrial activities. For example, miR-155 is enriched in mitochondrial fractions from mouse liver, and levels are increased following streptozotocin treatment, which induces type I diabetes and impairs mitochondrial function [17]. Highly purified hippocampal mitochondria were found with several miRNAs such as miR-146a, miR-142-3p, miR-142-5p under normal physiological conditions [18]. Similarly, miR-696 is known to regulate mitochondrial biogenesis and fatty acid oxidation in myocytes by targeting Peroxisome proliferator-activated receptor gamma coactivator 1-alpha (PGC-1*α*) while also leading to increased lipid deposition and decreased mitochondrial content in C2C12 cells [19].

Thus, mito-miRs may impact mitochondrial functioning in several ways; (i) by inhibiting mitochondrial translation, (ii) by stimulating a compensatory synthesis of an alternative strand of the mitochondrial genome, (iii) increasing compensatory mitochondrial transcription, (iv) may impact overall mitochondrial genome copy number [20] (v) regulating mitochondria-associated transcriptional factors activity [21, 22] and (vi) regulating various mechanisms involved in mitochondrial transcription and epigenetic modification though it remains controversial [23]. Recent reports have indicated the presence of Argonaute 2 (AGO2) inside the mitochondria raising prospects of the presence of RNA Induced Silencing Complex (RISC) like machinery inside the mitochondria [24]. A recent study by Fan et al. [25] describes the functional role of mito-miRs for the first time. The study showed the role of mitomiR-2932 in regulating mtDNA transcription and metabolism.

The current study presents for the first time mitochondria specific function of miRNA let-7a, which is found to be involved in targeting the expression of mitochondrial NADH dehydrogenase subunit 4 (ND4), in turn, inducing metabolic reprogramming in the early stages of breast cancer development.

## Results

### Let-7a is differentially localized inside the mitochondria of MCF 7 cells upon doxorubicin treatment

Doxorubicin (DOX) is known to induce mitochondrial stress by altering cell signaling, mitochondrial specific ROS generation, membrane potential alteration as well as impacting mitochondria specific localization of critical proteins [26]. Therefore, for detecting mito-miRs inside the mitochondria and to ensure specificity of their localization, we chose DOX to induce mitochondrial stress and understand the organelle-specific localization of various small RNAs.

**Fig. 1:**
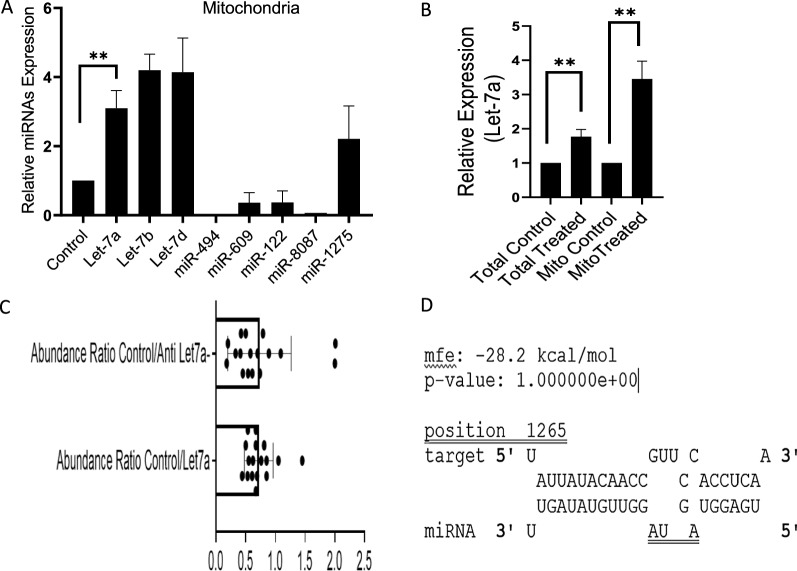
**A** Differential distribution of mitomiRs upon DOX induced mitochondrial stress in MCF-7 cells. The relative expression of the represented mitoRs was normalized by U6. **B** Relative expression of let-7a in cellular and mitochondrial RNA isolated from MCF-7 cells. **C** Mass spectrometric analysis of mitochondrial proteins in let-7a mimic/antisense transfected MCF-7 cells. Results are represented as abundance ratio control/let-7a mimic/antisense. **D** Predicted target site for let-7a in the ND4 3’-UTR. The p-value was calculated with the download version of RNAhybrid. The data are represented as mean + SD analyzed by unpaired t-test, n = 3. (p < 0.05). ** indicates the level of significance

Mitochondria were isolated from control, and DOX treated MCF-7 cells as described under material and methods, and purity was verified by western blotting and qPCR (Additional file 1: Fig. S1). Mitochondria were then processed for RNA isolation, quality analysis, and small RNA library generation that was then sequenced using Ion Torrent Proton™ System (Thermofisher) and analyzed. NGS data showed the differential distribution of different miRNAs inside the mitochondria, including miRNAs that were not reported previously (miR-8087, miR-5585-5p, miR-5585-3p, miR-7110, miR-8086, miR-567, miR-378a, miR-151a). To validate the NGS data, our RT-qPCR results also show altered expression of miRNAs in dox treated mitochondria of MCF-7 cells (Fig. 1A). The increased levels of let 7a, let 7b and let 7d were found upon treatment with DOX. Let-7a in particular, attracts our attention because of its recently revealed role in the metabolic reprogramming of cancer cells [27]. We performed RT-qPCR analysis of let-7a in cellular and mitochondrial RNA upon DOX treatment and observed approximately fourfold increase inside the mitochondria (Fig. 1B). Next we asked was how let-7a may influence the protein component encoded by mitochondrial genome. We transfected MCF-7 cells with let-7a antisense as well as a mimic and performed high throughput proteomic analysis for purified mitochondrial fractions. The results showed the altered abundance of nuclear-encoded mitochondrial proteins upon let-7a knockdown/overexpression (Fig. 1C). Since the impact of mito-miR let-7a on protein expression might be secondary in nature, we performed manual alignment studies using the NCBI BLAST tool to look for let-7a specific targets, and interestingly we discovered that 3’ UTR of ND4 gene harbours putative let-7a target site (Fig. 1D).

### Let-7a targets mRNA of mitochondrial gene ND4 (MT-ND4)

**Fig. 2:**
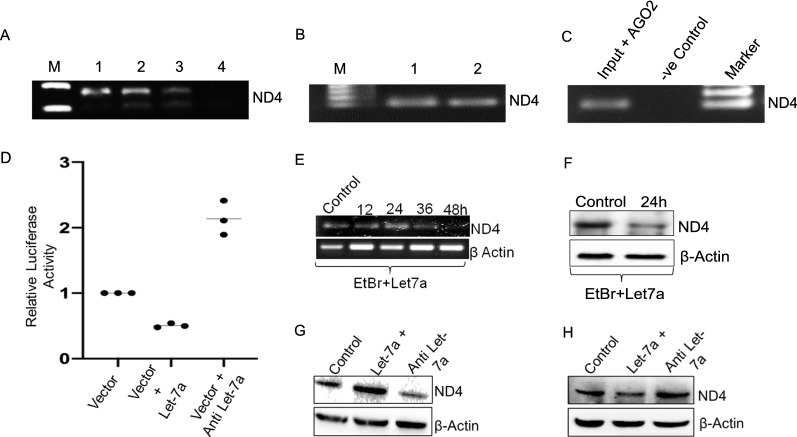
Validation of interaction between let-7a and ND4. **A** RT-PCR showing pulldown of ND4 with biotin labelled let-7a in cellular system of MCF-7 cells. M-marker, *Lane 1:* Biotin labelled let-7a and 300 µg of cellular lysate were incubated for 1 h and then magnetic streptavidin beads were added and incubated for another 1 h followed by RNA isolation and RT-PCR analysis, *Lane 2:* Biotin labelled let-7a and magnetic streptavidin beads were incubated for 1 h then lysate was added for another 1 h. *Lane 3:* Biotin labelled let-7a, magnetic streptavidin beads and lysate were added at the same time and incubated for 2 h followed by RNA isolation and RT-PCR analysis. **B** RT-PCR showing pulldown of ND4 in MCF-7 cells transfected with biotin labelled let-7a (20 nM). *Lane 1:* Biotin labelled let-7a and 300 µg of cellular lysate were incubated for 1 h and then magnetic streptavidin beads were added and incubated for another 1 h followed by RNA isolation and RT-PCR analysis, *Lane 2:* Biotin labelled let-7a and magnetic streptavidin beads were incubated for 1 h then lysate was added for another 1 h. **C** ND4 levels in RNA immunoprecipitation complex was analysed by RT-PCR and its negative control. **D** Relative activity of Luciferase in let-7a transfected MCF-7 cells using pMIR REPORT vector system. Let-7a significantly inhibit the translation of luciferase by directly binding to target site on ND4. **E** RT-PCR analysis showing half-life of ND4 transcript after combined treatment of EtBr and let-7a mimic in MCF-7 cells at different time points (12, 24, 36 and 48 h). **F** MCF-7 cells were treated with EtBr and transfected with let-7a mimic for 24 h. ND4 levels were analysed by Western blot analysis. **G** MDA-MB-231 cells were transfected with mimic and antisense of let-7a for 24 h. ND4 levels were determined by Western blot analysis (**H**) ND4 expression in MCF-7 cells. β-Actin is used as a loading control

After confirmation of a potential partial target site in 3’ UTR of ND4 gene, RNA pull-down experiments were designed to resolve the mRNA-miRNA interaction using biotin-labelled let-7a. Cellular fractions were used to isolate total RNA, followed by incubation with biotin-labelled let-7a and pull-down using magnetic streptavidin beads, followed by RNA isolation and RT-PCR for the ND4 gene. The results confirmed the let-7a-ND4 interaction (Fig. 2A). Furthermore, we transfected MCF-7 cells with biotin-labelled let-7a followed by whole-cell lysate preparation and performed the same pull-down, which again showed ND4 mRNA interaction with let-7a (Fig. 2B). The catalytic centre for miRNA-mediated silencing machinery is AGO2 that has been recently reported to be present inside the mitochondria [28]. To further validate if let-7a can directly target ND4 mRNA, we performed RNA immunoprecipitation which showed interaction of ND4 as well as let-7a with AGO2 (Fig. 2C). We then designed an indirect Luciferase assay to ascertain whether ND4 3′UTR is a direct target of let-7a. The potential target site was cloned in pMIR REPORT vector and co-transfected in MCF-7 cells with let-7a mimic and antisense followed by luciferase assay. The results indicate that let-7a overexpression leads to decreased luciferase activity while knockdown resulted in higher activity, indicating that the potential site is indeed targeted by let-7a (Fig. 2D).

Our results indicate that let-7a targets the 3′UTR of ND4 mRNA and regulates its expression. Therefore, we next sought to investigate whether the mechanism of regulation of ND4 expression by let-7a, is mRNA degradation or inhibition of translation. Ethidium bromide (EtBr) was used as mitochondria transcriptional inhibitor [29] in the presence of let-7a at different time points, followed by RT-PCR analysis of ND4 expression. The results showed that let-7a overexpression reduced the half-life of ND4 mRNA transcript from 36–48 h to 24–36 h (Fig. 2E). Further western blotting was also performed after blocking mitochondrial transcription, which showed that ND4 protein expression was down-regulated upon let-7a overexpression (Fig. 2F). These results thus indicate that let-7a directly targets ND4 mRNA leading to its decreased stability in mitochondria of MCF-7 cells. However, upon replication of the same experiment in another breast cancer cell line, MDA-MB-231, we got contradicting results showing let-7a overexpression leads to higher ND4 expression (Fig. 2G), whereas lower expression in MCF-7 cells was observed (Fig. 2H).

**Fig. 3:**
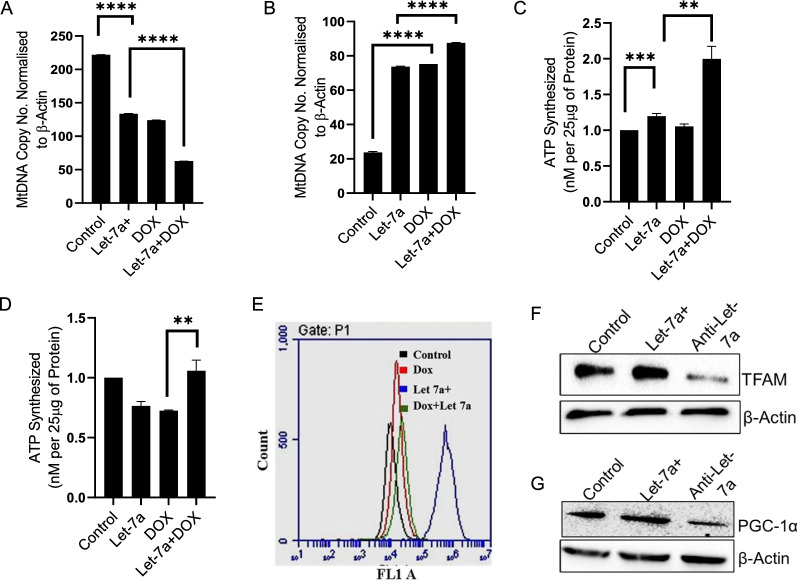
Effect of let-7a mimic and dox on mitochondrial and cellular parameters in MDA-MB-231 and MCF-7 cells. RT-qPCR was used to determine mtDNA level in MDA-MB-231 and MCF-7 cells. mtDNA/nDNA ratio represent mtDNA number in **A** MDA-MB-231, and **B** MCF-7 cells. ND4 used for mitochondrial gene was normalised to nuclear β-Actin. Effect of dox and Let-7a on cellular ATP levels in **C** MDA-MB-231 and **D** MCF-7 cells. **E** Flow cytometric analysis of mitotracker green in MCF-7 cells. **F** and **G** MCF-7 cells were transfected with mimic/antisense of let-7a for 24 h. Western blot analysis showing expression levels of mitochondrial biogenesis markers TFAM (28KDa) and PGC-1α (100KDa). The data are represented as mean + SD, analyzed by unpaired t-test, n = 3. (p < 0.05). (**p < 0.01), (***p < 0.001), (****p < 0.0001)

Since let-7a showed contradictory function in low versus high-grade breast cancer cell lines (MCF7 versus MDA MB 231), so we proceeded to evaluate various mitochondrial parameters, which might be differentially regulated by let-7a. Both the cell lines were treated with let-7a overexpression or doxorubicin, and both were followed by evaluation of mtDNA content using qPCR. MDA-MB-231 cells showed decreased mtDNA copy number in all the treatments compared to control cells (untreated) (Fig. 3A). MCF-7 cells showed increased mtDNA content upon the individual as well as combined treatment indicating that mitochondria indeed is involved in the anticancer response of let-7a and DOX in MCF-7 cells; they try to induce mitochondrial normalcy and in turn, metabolic reprogramming or cell death (Fig. 3B). The existing literature suggests that MDA-MB-231 relies heavily on glycolysis as means of energy production and is highly metastatic. MCF-7 cell line, on the other hand, represents low-grade non-metastatic breast cancer and depends more on OXPHOS for energy production [30]. Thus, the contrasting results we obtained in these experiments point out that at early stages of cancer progression, let-7a tries to interfere with mitochondrial central dogma while the same is not true in advanced stages of cancer. To further understand the contrasting role of let-7a in both the cell lines, we calculated ATP output in both the cell lines. The results showed that individual let-7a treatment in MDA-MB-231 cells showed a slight increase in ATP level, whereas MCF-7 cells showed about 20% reduction in ATP output (Fig. 3C and D), indicative of an OXPHOS inhibitory role of let-7a. Since ND4 is an integral part of OXPHOS; this result is in agreement with our earlier data showing ND4 as a target of let-7a.

### Let-7a impacts mitochondrial mass and biogenesis in MCF-7 cells

After establishing a definitive role of let-7a in mitochondrial functioning in MCF-7 cells, we further sought to understand the impact of let-7a on mitochondrial mass, biogenesis, and other parameters. Cells were transfected with let-7a followed by an analysis of relative mitochondria numbers using mitotracker dye. The results showed a significant increase in mitochondria numbers following let-7a as indicated by flow cytometer (Fig. 3E) and confocal microscopy data (Additional file 1: Fig. S2A). Furthermore, western blot analysis also showed increased expression of PGC-1α and TFAM upon let-7a overexpression (Fig. 3F and G). Both of which indicate the induction of mitochondrial biogenesis, and therefore, let-7a may have the capability to induce mitochondrial biogenesis at the cellular level. Similarly, an apoptotic profile of let-7a overexpressed cells following Annexin/PI staining using flow cytometer revealed severe apoptosis in response to combined treatment of DOX and let-7a as compared to either DOX or let-7a alone. The data indicate that DOX when combined with let-7a, shifts more cells towards late apoptosis (Additional file 1: Fig. S2B and S2C). Furthermore, H_2_DCFDA staining in MCF-7 cells showed increased ROS species in the combined treatment of let-7a and dox as compared to individual treatments.

### Let-7a target ND4 expression and alters oxidative phosphorylation in MCF-7 cells

**Fig. 4:**
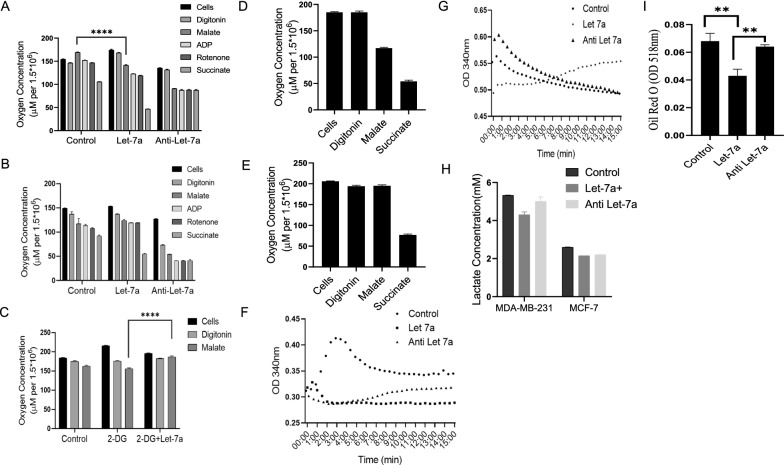
Measurement of oxygen concentration using Oroboros O2K Respirometer. Representative graphs showing measurement of oxygen consumption rate upon addition of different substrates and inhibitor in **A** MDA-MB-231 cells and **B** MCF-7 cells. **C** Measurement of oxygen concentration after combined treatment of 2-DG and let-7a in MDA-MB-231 cells. Bar graphs represents oxygen concentration in ρ^0^ cells of **D** MDA-MB-231 and **E** MCF-7 cells. Measurement of mitochondrial complex I activity in let-7a transfected (mimic/anti) MDA-MB-231 and MCF-7 cells. Data is represented as absorbance versus time. **F** Showing Complex I activity in MDA-MB-231cells and **G** MCF-7 cells. **H** Measurement of lactate levels in MDA-MB-231 and MCF-7 cells**.** Let-7a showing inhibition of glycolysis in MDA-MB-231 cells. Effect of let-7a on adipogenesis. **I** Graphs representing staining of Oil Red O in MCF-7 cells transfected with mimic/antisense of let-7a. Level of significance was tested by Two-way anova in Fig A, B and C, (****p < 0.0001). The data are represented as mean + SD analyzed by unpaired t-test, n = 3. (p < 0.05). (**p < 0.01)

After establishing ND4 as a target of let-7a, we were interested in exploring the impact of the interaction on mitochondrial-mediated metabolism in both cell lines. The difference in mtDNA and ATP level in low (MCF-7) and high (MDA-MB-231) metastatic breast cancer cell lines was observed upon let-7a overexpression. We next determined the regulation of ND4 at the protein level in both the cell lines. Whereas let-7a over-expression resulted in increased ND4 protein levels in MDA-MB-231 cells and reduced ND4 levels were observed in MCF-7 cells, indicating a regulatory role of let-7a in a cell-specific manner. Further, we determined the impact of let-7a on mitochondrial activity in MDA-MB-231 and MCF-7 cells by measuring oxygen concentration using the Oroboros O2K respirometer. We found that in MDA-MB-231 cells, the addition of malate does not stimulate Complex-I in control cells, whereas in the presence of let-7a, significant induction of respiration was observed as represented by reduced oxygen concentration. The addition of ADP and inhibitor of Complex-I rotenone was followed by malate, and the results showed a slight decrease in oxygen concentration, whereas, in let-7a transfected cells, rotenone stabilizes the oxygen concentration, indicating less dependency on Complex-I of MDA-MB-231. Further to confirm the regulation of Complex-I by let-7a, Complex-II was stimulated after the addition of succinate, and interestingly, a significant decline in oxygen concentration was observed whereas, in the presence of let-7a antisense, the effects were reversed (Fig. 4A). Further, the same experiment was performed using MCF-7 cells, and the results showed that the addition of malate does not induce respiration as compared to control cells, whereas the addition of rotenone stabilizes oxygen concentration, and upon injection of succinate, there was a significant reduction of oxygen concentration (Fig. 4B). Altogether, it demonstrates that let-7a regulate MDA-MB-231 and MCF-7 cells differently, indicating MDA-MB-231 relying less on OXPHOS, whereas MCF-7 cells are more dependent on OXPHOS to fulfil their energy requirement.

Further, to confirm the metabolic phenotype of MDA-MB-231 cells and the impact of let-7a, we treated the cells with 2-deoxy-D-glucose (2-DG), which was later combined with a mimic of let-7a. 2-DG is a known glycolytic inhibitor that inhibits glycolysis by phosphorylating the hexokinase II (HKII). The recorded result showed that the addition of malate after permeabilization does not induce respiration in 2-DG treated cells as represented by increased oxygen concentration when compared to untreated cells. On the other hand, in cells with combined treatment of 2-DG and let-7a, after the addition of malate, let-7a masks the inhibitory effect of 2-DG and stimulates Complex I to generate ATP as indicated by a moderate decrease in oxygen concentration. The high dependency of MDA-MB-231 cells on glycolysis was inhibited by 2-DG, whereas the presence of let-7a shift the cells towards OXPHOS (Fig. 4C). The metabolic difference in both the cell lines was also verified by generating ρ^0^ cells of MDA-MB-231 and MCF-7 cells. The results show that the addition of the malate in MDA-MB-231 cells stimulates respiration irrespective of the deleted mtDNA **(**Fig. 4D)**,** whereas, in MCF-7 cells, malate does not have any effect on respiration (Fig. 4E). It was speculated that the depletion of mtDNA does not affect the energy-generating process in MDA-MB-231 cells; however, MCF-7 cells are not able to generate energy in the absence of mitochondria, indicating their dependence on mitochondria to meet energy demands. Therefore, the results confirm the difference in metabolic phenotypes of the two cell types.

To further confirm the metabolic adaptations in both cell lines, we were curious to measure the Complex I activity directly in MDA-MB-231 and MCF-7 cells. The results showed lower activity of Complex I in untreated MDA-MB-231 cells, represented by increased absorbance, while in let-7a mimic transfected cells, absorbance decreases, indicating let-7a induces the Complex I activity (Fig. 4F). Whereas in let-7a mimic transfected MCF-7 cells, absorbance increases indicating let-7a inhibits the OXPHOS by reducing Complex-I activity (Fig. 4G). Cells transfected with antisense of let-7a showed an increase and decrease in absorbance in MDA-MB-231 and MCF-7 cells, respectively (both nearing to control). The obtained results are consistent with the measured mitochondrial respiration using Oroboros.

Lactate accumulation in breast cancer cells can serve as an additional source of energy and can activate gene expression to support tumor growth. As shown in our previous results, relative to MCF-7 cells, MDA-MB-231 cells rely less on OXPHOS. We were therefore interested in determining the amount of lactate produced in both cell lines after transfecting cells with mimic and antisense of let-7a. Lactate production was measured following let-7a transfection in MCF-7 and MDA-MB-231 using the Cayman assay kit. The results obtained confirmed that lactate concentration is higher in MDA-MB-231 control cells as compared to MCF-7 cells but the concentration decline in let-7a transfected cells indicating an inhibitory effect of let-7a towards glycolysis. Conversely, lactate accumulation was observed in cells after transfection with let-7a antisense in MDA-MB-231 cells. In MCF-7 let-7a transfected cells, the minute but significant change in lactate concentration was observed as compared to control cells (Fig. 4H).

Previous results showed let-7a mediated metabolic reprogramming; we further explore its role by quantitatively measuring the lipid droplets in MCF-7 cells in the presence of mimic and antisense of let-7a. It was evidenced that high-fat accumulation was associated with adverse cancer survival as high fat along with higher expression of metabolic genes lead to increased cancer cell proliferation [31]. Here, we observed that let-7a overexpression inhibits fat accumulation in cancer cells as indicated by a decrease in absorbance as compared to control cells, whereas in the presence of antisense of let-7a absorbance is similar to control (Fig. 4I).

## Discussion

The present study explores the possible regulatory role of miRNAs present inside the mitochondria. Using high throughput NGS technology, we found that mito-miRs are differentially localized in MCF-7 cells when exposed to mitochondrial stress (DOX), indicating that presence of miRNAs inside the mitochondria is not a random but a rather well-coordinated effort. The most interesting of all the mitochondrial localized miRNAs found and validated in our study was the tumor suppressor miRNA let-7a. The presence of let-7a inside the mitochondria provides evidence about its potential role in regulating mitochondrial functions. Several computational methods have been designed to determine the target site of miRNAs; however, (none of these methods includes the mitochondrial genome specifically) [32]. It has been reported that full complementarity is not the criteria for miRNAs mediating their action, as few studies reported the regulation of gene expression at the transcriptional level [33]. Using the BLASTn tool, we found that 3′ UTR of ND4 has a putative partial binding site for let-7a. Besides other functions, let-7a was recently reported in regulating metabolic reprogramming in cancer cells [27].

This interaction was confirmed using RNA-RNA and RNA–protein interaction studies showing that let-7a binds to 3′UTR of ND4 gene and both of these RNA molecules interact with AGO2, a critical player of miRNA mediated mRNA targeting machinery [34, 35]. The results obtained using RNA immunoprecipitation assay were confirmed by Luciferase reporter assay in the presence of mimic/antisense of let-7a, which again showed the downregulation of ND4 expression upon overexpression of let-7a. Further, our results suggest a post-transcriptional inhibition of ND4 mRNA by let-7a, which is consistent with the principle of nuclear/cellular mechanisms by miRNAs. In a series of experiments, let-7a was observed to regulate mitochondrial parameters, which were further supported by the role of let-7a in the induction of mitochondrial biogenesis in MCF-7 cells. Although, we did not observe any impact of let-7a on overall mitochondrial translation regulation of ND4 expression at post-transcriptional levels and other related parameters indicating mitochondrial entry of miRNA is not a random event. Though the mutation status of the ND4 gene was not checked in both the cell lines during the study but SNPs database analysis revealed the absence of any mutation in the sequence with a potential let-7a binding site.

Altered metabolism and bioenergetics are the hallmarks of cancer involved in promoting cancer progression through mediating metastasis, the microenvironment, and in development of chemoresistance. Different studies have reported that cancer cell metabolism is not stable, and reprogramming of the mitochondrial metabolism pathways is consistently involved in tumor progression, including breast cancer [36]. Thus, novel targeted approaches need to be devised to treat metabolic alterations. Our study demonstrates inhibition of OXPHOS by let-7a in MCF-7 cells, which was supported by increased lactate concentration, decreased Complex I activity, and inhibition of adipogenesis, indicating the involvement of let-7a in mitochondrial metabolism while contrasting results were obtained in MDA-MB-231 cells.

In conclusion, our study demonstrates for the first time that let-7a enters into the mitochondria where it post-transcriptionally represses ND4 expression resulting in altered Complex I activity. In MCF-7 cells, let-7a decreases oxidative phosphorylation whereas, in MDA-MB-231 cells, let-7a reprogram metabolism via OXPHOS that is strictly a cell-specific phenomenon (happens in MCF-7 but not in MDA MB 231 cells). This indicates that let-7a entry to mitochondria in MCF-7 cells perhaps may lead to a metabolic switch forcing cells to look for alternative energy sources and metabolic reprogramming, resulting in altered phenotype.

## Material and methods

### Cell culture and transfection

Breast cancer cell lines were procured from Animal Type Cell Culture (ATCC) and National Centre for Cell Science, Pune (NCCS). Two models of breast cancer cells having different metastatic potential that is MDA-MB-231 and MCF-7 cells were grown in Dulbecco's Modified Eagle's Medium (DMEM, Gibco, Invitrogen, USA) supplemented with 1% penicillin, streptomycin (PS) antibiotic solution (Gibco, Invitrogen, USA) and 10%(v/v) heat-inactivated fetal bovine serum (FBS) (Gibco, Invitrogen, USA) in CO_2_ incubator at 37 °C [30]. Cells were treated with 50 nM DOX and transfected with 20 nM of mimic and antisense of let-7a using Lipofectamine 2000 (Invitrogen) [37].

### Generation of ρ^0^ cells

ρ^0^ cells are the cells that have depleted mtDNA generated by exposing the cells with EtBr in the presence of uridine. MCF-7 and MDA-MB-231 cells were seeded in culture medium containing EtBr (50 µg/ml) supplemented with uridine (50 mg/ml) for 15 days. After completion of the incubation period, cells were harvested and processed as per the experimental requirement [3].

### Mitochondria isolation and purity analysis

After completion of subsequent time point of cell culture and treated cells were harvested, and mitochondria were isolated using Mitochondria Isolation Kit from Cultured Cells (ThermoScientific™). Briefly, 5*10^6^ cells were harvested in mitochondrial isolation buffer A followed by mitochondrial isolation reagent B. Mixture was then centrifuged at 700*g* for 10 min at 4 °C. After fractionation, the supernatant containing cytoplasmic fraction was stored and pellet containing mitochondrial fraction were washed with reagent C followed by RNAse treatment (10 mg/ml; 10 µl per 5 million cells) for 30 min at RT and then used for different parts of the experiment. The purity of the mitochondrial fraction was analyzed by Immunoblotting and RT-PCR for genes associated with mtDNA.

### RNA isolation and sequencing

Cellular and mitochondrial RNA was extracted using TRIZOL reagent (Invitrogen) followed by purity analysis and RNA quantification by Nanodrop™ 2000 Spectrophotometer [37]. After quantification, 5 µg of each RNA sample were processed for, small RNA sequencing using Ion Torrent Proton™^.^ (Thermofisher).

### Reverse transcription and quantitative real-time PCR

To determine the relative expression of different genes, RT-PCR/qPCR was performed. For miRNA specific quantification, MMLV Reverse transcriptase was used to reverse transcribe 200 ng of RNA. For RT-qPCR, the reaction mixture was prepared as following; 200 nM of forward and reverse primers were added, followed by 2 µl of diluted cDNA, 5 µl of 2X SYBR green master mix and then nuclease-free water was added to make a final reaction mixture of 10 µl. Initially, the reaction was initiated at 95 °C for 10 min, denaturation for 10 s followed by annealing and extension at 58 °C and 72 °C, respectively. The relative expression of the mitomiRs was normalized by U6 expression and calculated by the threshold cycle method (2^−^^CT^) [38]. Primers used for miRNAs, mitochondrial and nuclear specific genes are provided below:

**Table:** 

Primer	Sequence
Let-7a-Forward	TATACAACCTACTACCTCA
Let-7a-mimic	UGAGGUAGUAGGUUGUAUAGUU
Let-7a-antisense	ATATGTTGGATGATGGAGT
Let-7a-Forward	UGAGGUAGUAGGUUGUAUAGUU(Btn)
Let-7b-Forward	CATACAACCTACTACCTCA
Let-7b-cDNA	GTTGGCTCTGGTGCAGGGTCCGAGGTATTCGCACCAGAGCCAACAACCA
miR-494-cDNA	GTTGGCTCTGGTGCAGGGTCCGAGGTATTCGCACCAGAGCCAACAGAGA
miR-494-Forward	GTAGGTTGTCCGTGTTGTC
miR-609-cDNA	GTTGGCTCTGGTGCAGGGTCCGAGGTATTCGCACCAGACCCAACAGAGA
miR-609-Forward	GTTGGAGGGTGTTTCTTCTC
ND4-Forward	TTTTTAACCAAATCAACAACAACC
ND4-Reverse	TGTGAGGGGTAGGAGTCAGG
ND1-Forward	ATGGCCAACCTCCTACTCCT
ND1-Reverse	GGGCCTTTGCGTAGTTGTAT
β-Actin-Forward	AGAGCTACGAGCTGCCTGAC
β-Actin-Reverse	AGCACTGTGTTGGCGTACAG
U6-Forward	GCTTCGGCAGCACATATACTAA
U6-Reverse	AACGCTTCACGAATTTGCGT

### Proteomic analysis

The differential expression of proteins expressed in cells was determined using mass spectrometric (MS) based proteomics. Mitochondria were isolated from the cells transfected with mimic and antisense of let-7a. The mitochondrial pellet was lysed in lysis buffer containing 6 M guanidium chloride, Tris (pH-8.5) − 0.1 M, and kept at 90 °C for 10 min. The lysate was then exposed to sonication for breaking nucleic acid and reducing the viscosity of shiny material and again kept at 90 °C for 10 min. Centrifugation was carried out at 15000K for 20 min at room temperature, and the supernatant was collected and then, protein concentration was estimated using Bradford reagent. Approximately 2 mg/ml of lysate was further processed for MS analysis. MS analysis of peptide mixtures was performed using EASY-nLC 1000 system (ThermoFisher Scientific) coupled to Thermo Fisher-QExactive equipped with nanoelectrospray ion source. 1.0 µg of the peptide mixture was resolved using a 25 cm PicoFrit column filled with 1.8 µm of C18-resin. The peptides were loaded with buffer A and eluted with a 0–40% gradient of buffer B (95% acetonitrile, 0.1% formic acid) at a flow rate of 300 nl/min for 10 min and MS data was acquired.

### Biotinylated pulldown assay

Biotinylated pull-down assay was performed using biotin-labeled let-7a. MCF-7 cells were harvested and the cellular lysate was prepared using lysis buffer (Tris pH 7.4-50 mM, NP40-1%, NaCl-150 mM, DTT-1 mM, and PI cocktail). Approximately 300 µg of lysate was incubated with biotinylated let-7a and magnetic streptavidin beads (Sigma-Aldrich) in RNA protein binding buffer (Tris pH 7.8–0.2 M, NaCl-0.5 M, MgCl_2-_ 20 mM and Tween20-1%) at 4 °C. In total, the mixture was incubated for 2 h to explore the expected interaction of let-7a with the mitochondrial genome.

Biotinylated pull-down assay was also performed after transiently transfecting MCF-7 cells with biotinylated let-7a (20 nM) for 24 h, and then lysate was prepared using the above-described lysis buffer. After completion of the incubation time, a magnetic frame was used to remove the supernatant, and magnetic beads containing complex was collected for RNA extraction, and RT-PCR was performed to assess the binding of let-7a with the target [25].

### RNA immunoprecipitation assay

RNA immunoprecipitation was performed using Dynabeads Protein A (Novex, Life Technologies) and anti-AGO2 antibody (Sigma). Briefly, after removing media from the cultured MCF-7 cells, cells were washed with 1× PBS and then crosslinked using 2% paraformaldehyde for 5 min. Crosslinking was stopped by adding 125 mM glycine and lysed using lysing buffer (Tris pH-50 mM, NaCl-150 mM, MgCl_2_-20 mM and NP40-0.8%, glycerol-5%, EDTA-10 mM and SDS-0.5%) with frequent tapping on ice for 45 min and then centrifuged at 10,000 rpm for 5 min at 4 °C. Approximately 250 µg of lysate was incubated with an anti-AGO2 antibody with continuous mixing at 4 °C followed by the addition of Dynabeads Protein A for another 1 h. A magnetic frame was applied to remove the supernatant, and beads containing immunocomplex were washed with lysis buffer. Reverse crosslinking was achieved by adding 5 µl of 5 M NaCl per 100 µl solution at 65 °C then RNA was isolated using the TRIzol method, and then RT-PCR was performed for analyzing the expression of ND4 [39].

The expression of ND4 was assessed to confirm its interaction with AGO2.

### Luciferase assay

The pMIR-REPORT miRNA Expression Reporter Vector System was used to analyze the interaction of let-7a with the target sequence (MT-ND4) [40]. Let-7a targeting sequence was commercially synthesized from Sigma and cloned into HindIII restrict endonuclease of pMIR Report vector. Vector was co-transfected in MCF-7 cells with mimic and antisense of let-7a. 24 h of post-transfection cells were harvested and lysed using glycerol containing lysis buffer, and then lysate was mixed with buffer containing luciferin and luminescence was measured using Promega Glo Max. The expression was normalized with protein concentration.

### ND4 mRNA stability assay

The effect of let-7a on mtDNA encoded transcript stability was evaluated by treating cells with mitochondrial transcriptional inhibitor EtBr at a concentration of 50 ng/ml. Cells were treated with EtBr for 24 h, followed by transfection of let-7a mimic for another 24 h. Cells were than harvested for analyzing the expression of ND4 using reverse transcriptase PCR.

### Immunoblotting

Further, to determine the half-life of mtDNA encoded ND4, cells were treated with EtBr and transfected with a mimic of let-7a for 12, 24, 36, and 48 h. Cells were harvested for RNA isolation and processed for RT-PCR. The reaction was initiated at 94 °C for denaturation for 10 min followed by annealing and extension at 62 °C (30 s) and 72 °C (30 s), respectively, for 35 cycles.

### MtDNA copy number analysis

Briefly, DNA was isolated from let-7a transfected (mimic/antisense), and DOX treated MCF-7 and MDA-MB-231 cells, using phenol–chloroform method. Approximately 50 ng total DNA was used for quantitative analysis of mtDNA level by qPCR using SYBR Green master mix. The relative copy number of mtDNA was determined using mitochondrial-specific primer for ND4 and nuclear specific β-Actin. Quantitative analysis was carried out using the following reaction conditions; the pre-amplification step was carried out at 95 °C for 3 min, followed by 40 cycles at 95 °C for 10 s, annealing was performed at 58 °C for 30 s and final extension was carried out at 72 °C for 30 s. After obtaining the Ct values, the mtDNA copy number was calculated using the following formula: Copies of mtDNA = 2 * 2^^Ct^ [41].

### Measurement of cellular ATP

Total ATP level was measured in MDA-MB-231 and MCF-7 cells, after 24 h of transfection with let-7a mimic/antisense (20 nM) and treatment with DOX. Cells were harvested and an adequate amount of lysis buffer (Tris–Cl pH 7.8–25 mM, EDTA 2 mM, glycerol 10%, Triton X 100-1%) were added then samples were boiled for 5 min at 95 °C, and protein concentration was determined using Bradford reagent. All the components used in ATP assay are procured from Invitrogen. The mixture containing lysate and reaction buffer were incubated on ice for 10 min in the dark and then luminescence was recorded using Glo max luminometer (Promega) [42].

### Estimation of the number of mitochondria

Mitotracker red (Invitogen™, M22426) was used to determine the number of mitochondria present in DOX treated and let-7a transfected (mimic/antisense) MCF-7 cells. Briefly, cells were seeded on a coverslip and treated as described. After 24 h of treatment, media was removed and cells were washed with pre-warmed culture media and then incubated with media containing 300 nM of mitotracker red (Molecular Probes, Thermo) for 40 min in a CO_2_ incubator at 37 °C. After incubation, mitotracker media was removed and cells were washed with pre-warmed media, and cells were fixed with 3.7% paraformaldehyde prepared in complete growth media for 15 min at 37 °C and then washed three times with 1× PBS. Cells were permeabilized using permeabilization buffer (0.2% Triton X-100) for 15 min at 37 °C and then cells were again washed with 1× PBS. Finally, cells were stained with DAPI diluted in PBS for 20 min and then washed three times with 1×PBS. Using Antifade mounting media (Sigma), coverslips were mounted on clean microscopic glass slides and images were captured using Olympus Fluoview FV1000 [43, 44]. The same analysis was also performed using flow cytometry.

### Measurement of ROS levels

H_2_DCFDA fluorescent probe was used to determine intracellular ROS. ROS levels were measured in let-7a transfected (20 nM mimic/antisense) and DOX treated (50 nM) MCF-7 cells after incubating with 5 µM H_2_DCFDA (Invitrogen, Molecular Probe) for 30 min in the dark. After incubation, cells were washed with 1× PBS and collected for flow cytometer analysis. For the analysis of each sample, 50,000 events were captured, and data were analyzed [37].

### Protein extraction and western blot analysis

MCF-7cells were treated and transfected with the above-mentioned protocol and after 24 h of treatment and transfection, cells were harvested and washed with 1× PBS. Protein lysates were prepared using RIPA lysis buffer and quantified by using Bradford reagent. About 100 µg of protein sample were subjected to SDS-PAGE on 10% and 12% gel, which were then transferred onto the nitrocellulose membrane. The membranes were probed for overnight at 4 °C with primary antibodies PGC-1α (1:1000, Sigma), TOMM 22 (1:3000, Sigma), MT-ND4 (1:800, Sigma), TFAM (1:1000, Sigma), and β-Actin (1:3000, Sigma). After overnight incubation, membranes were subsequently washed three times with 1× PBST and incubated with HRP-conjugated secondary antibodies (1:10,000) (Invitrogen) for 2 h at room temperature. After completion of secondary antibody incubation, blots were rewashed and analyzed using ECL, and images were captured using Image 2.0 software from Biorad [37]. β-Actin was taken as a housekeeping gene.

### Annexin V/propidium iodide analysis

An apoptotic profile of MCF-7 cells determined after 24 h of transfection with mimic/antisense of let-7a and treatment with dox using flow cytometer. After completion of subsequent treatment time, cells were harvested and washed with 1× PBS followed by the addition of 5 µl Propidium iodide dissolved in 1× annexin binding buffer which was then resuspended in 80 µl of 1× annexin binding buffer. After that 5 µl of FITC Annexin V and 1 µl of PI (propidium iodide) was added to each sample and were incubated for 15 min at room temperature. After the incubation period, stained cells were processed and approximately, 50,000 events were captured using flow cytometer. Reagents used in this assay were purchased from Invitrogen™.

### Measurement of oxygen concentration

Measurement of mitochondrial respiration is essential to gain insight into mitochondria respiration capacity of complexes (I–IV) and metabolism and can be accomplished by measuring oxygen concentration using a high-resolution Oxygraph. To measure the respiration capacity of different complexes, different substrates and inhibitors can be employed. Respiration of permeabilized MDA-MB-231 and MCF-7 cells was investigated by substrate-uncoupler-inhibitor titrations (SUIT) protocol with slight modifications [45]. Approximately 1 * 10^6^ cells were added in the Oxygraph chamber containing serum-free DMEM media. 16 µl (8 µM) of digitonin was injected into the Oxygraph chamber containing cell suspension for permeabilization and respiration was recorded for 10 min. Malate and succinate at a concentration of (5 mM) and (10 mM) respectively were added in the presence of Complex I (CI) inhibitor rotenone to determine CI or CII linked respiration. ADP was added at 2.5 mM to obtain OXPHOS capacity. After adding substrates and inhibitors, respiration was recorded for 10 min each until the signal become stable.

### Assessment of glycolysis in MDA-MB-231 and MCF-7 cells

Cayman’s MitoCheck Complex I Activity assay kit (Item No. 700930) was used to detect the difference in Complex I activity of MDA-MB-231 and MCF-7 cells. Cells were seeded and transfected with 20 nM mimic and antisense of let-7a for 24 h and then cells were harvested and processed according to the manufacturer’s protocol.

### Assessment of glycolysis in MDA-MB-231 and MCF-7 cells

Approximately, 7000 cells per well were seeded in 96 well cell culture plates and then allowed the cells to grow overnight in CO_2_ at 37°. After incubation, cells were transfected with 20 nM mimic and antisense of let-7a for 24 h. After completion of time point, samples were processed as prescribed by manufactures instruction (Cayman Chemical, Glycolysis Cell-Based Assay Kit, Item No. 600450). At last absorbance was recorded at 490 nm using a microplate reader (Synergy™, Multimode Microplate Reader, BioTek) and concentration of lactate was measured using standard plotted for l-Lactate (mM).

### Oil Red O staining in cultured MCF-7 cells

Quantitative analysis of deposition of lipids was determined by Oil Red O staining. Briefly, cells were seeded in 24 well plate and transfected with 20 nM mimic/antisense of let-7a. After 24 h of transfection, media was discarded and fixed with 10% formalin for 5 min. After fixation, cells were subsequently washed with 60% isopropanol and then stained with a stock solution of Oil Red O (containing 3parts of Oil Red O and 2 parts of water) for 10 min. Cells were then washed with water and again incubated with 100% isopropanol for 10 min and OD was obtained at 518 nm and desired images were taken [46].

### Statistical analysis

MS Excel and Graphpad Prism were used to analyze the data and to plot the graphs. Each experiment was performed three times. The results are presented as mean ± SD. A two-tailed t-test and two-way ANOVA were used for testing the level of significance.

## Supplementary Information

**Additional file 1: Figure S1**. Isolation of mitochondria and its Purity Analysis. **(S1A)** The purified mitochondrial fraction was prepared from MCF-7 cells with and without DOX (50 nM) treatment and resolved on 12% SDS, transferred on nitrocellulose membrane and then probed for TOM22 (22KDa) and β Actin (42KDa); **(S2B)** Mitochondrial RNA was isolated and then amplified for any nuclear contamination using ND4 for mitochondria and β actin for nuclear fraction. **(S2C)** Purity was also confirmed by RT-qPCR; MT- Mitochondrial Treated; CC- Cytosolic Control; CT—Cytosolic Treated. **Figure S2**: Analysis of mitochondrial content. (**S2A**) Confocal microscopy analysis of mitochondrial biogenesis in MCF-7 cells. (**S2B)** Apoptotic profile after treating cells with dox and transfecting with mimic of let-7a, obtained after Annexin V/PI staining using flow cytometer. (**S2C)** Graphical representation of the number of cells in each quadrant. (S2D) Flow cytometric analysis showing ROS levels obtained after staining with H_2_DCFDA in MCF-7 cells.
