# Supplementary material for: Let-7a induces metabolic reprogramming in breast cancer cells via targeting mitochondrial encoded ND4
Source: Cancer Cell Int. 2021 Nov 27;21:629. doi: 10.1186/s12935-021-02339-3 (PMC8627041; doi:10.1186/s12935-021-02339-3)
Supplement: Supplementary file 1 — Additional file 1: Figure S1. Isolation of mitochondria and its Purity Analysis. (S1A) The purified mitochondrial fraction was prepared from MCF-7 cells with and without DOX (50 nM) treatment and resolved on 12% SDS, transferred on nitrocellulose membrane and then probed for TOM22 (22KDa) and β Actin (42KDa); (S2B) Mitochondrial RNA was isolated and then amplified for any nuclear contamination using ND4 for mitochondria and β actin for nuclear fraction. (S2C) Purity was also confirmed by RT-qPCR; MT- Mitochondrial Treated; CC- Cytosolic Control; CT—Cytosolic Treated. Figure S2: Analysis of mitochondrial content. (S2A) Confocal microscopy analysis of mitochondrial biogenesis in MCF-7 cells. (S2B) Apoptotic profile after treating cells with dox and transfecting with mimic of let-7a, obtained after Annexin V/PI staining using flow cytometer. (S2C) Graphical representation of the number of cells in each quadrant. (S2D) Flow cytometric analysis showing ROS levels obtained after staining with H2DCFDA in MCF-7 cells. [file 12935_2021_2339_MOESM1_ESM.docx]

**Supplementary Figure S1**

**
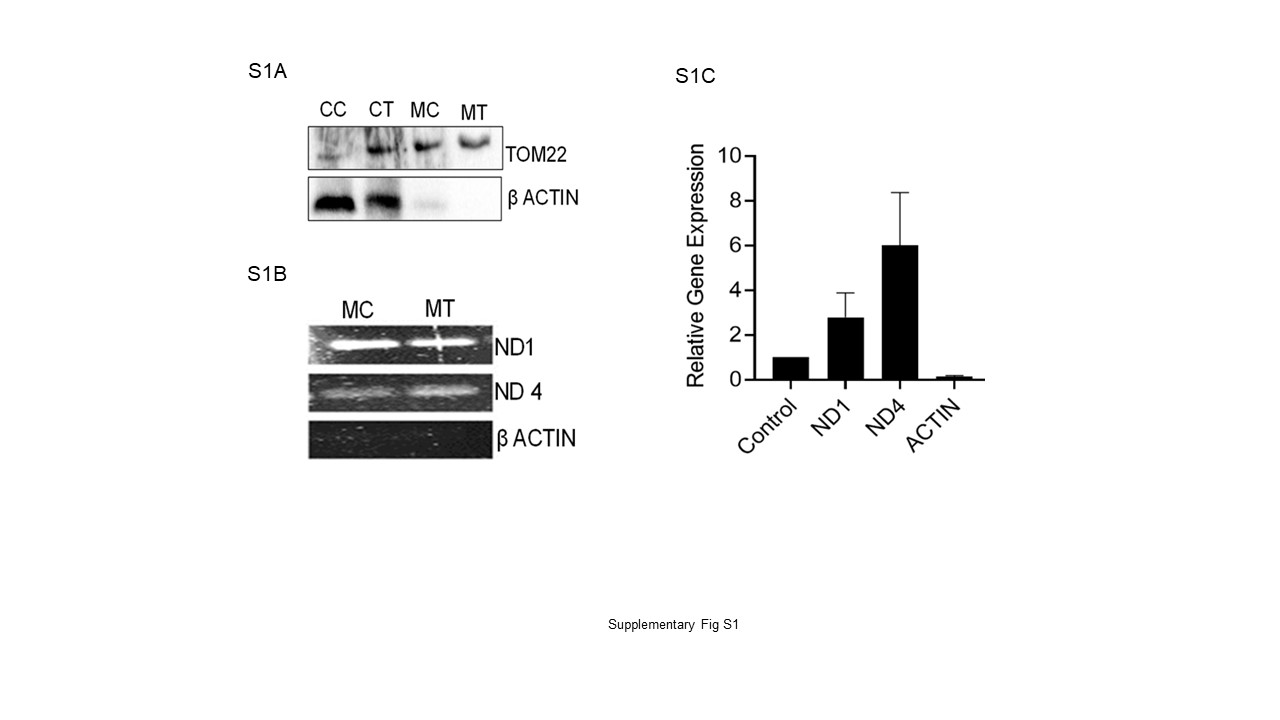
**

**Supplementary Figure S1**: Mitochondria Isolation and its Purity Analysis. **(S1A)** The purified mitochondrial fraction was prepared from MCF-7 cells with and without DOX (50nM) treatment and resolved on 12% SDS, transferred on nitrocellulose membrane and then probed for TOM22 (22KDa) and β Actin (42KDa); **(S2B)** Mitochondrial RNA was isolated and then amplified for any nuclear contamination using ND4 for mitochondria and β actin for nuclear fraction. **(S2C)** Purity was also confirmed by RT-qPCR; MT- Mitochondrial Treated; CC- Cytosolic Control; CT - Cytosolic Treated

**Supplementary Figure S2**

**
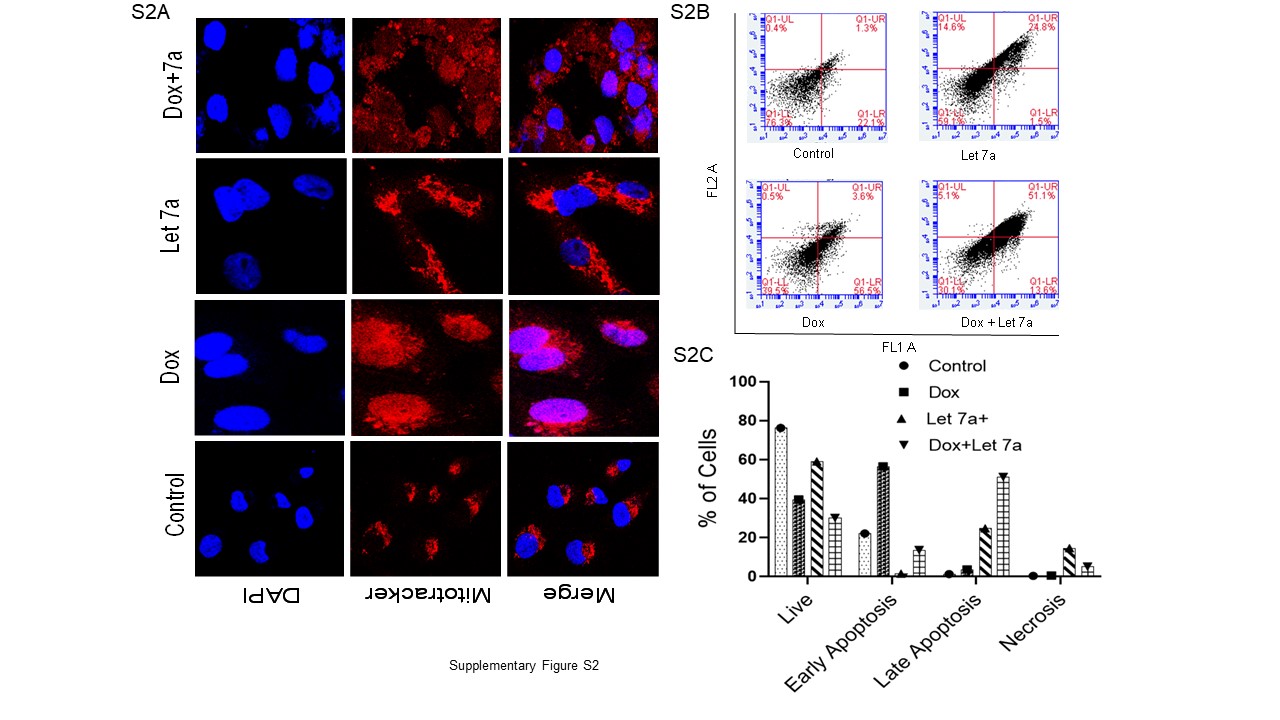
**

**Supplementary Figure S2**: Analysis of mitochondrial content. (**S2A**) Confocal microscopy analysis of mitochondrial biogenesis in MCF-7 cells. (**S2B)** Apoptotic profile after treating cells with dox and transfecting with mimic of let-7a, obtained after Annexin V/PI staining using flow cytometer. (**S2C)** Graphical representation of the number of cells in each quadrant.
